# Light-intensity Activity-related Nausea: An Unusual Presentation of Pheochromocytoma

**DOI:** 10.7759/cureus.5930

**Published:** 2019-10-17

**Authors:** Sakditad Saowapa, Parin Rattananon, Chutintorn Sriphrapradang

**Affiliations:** 1 Internal Medicine, Faculty of Medicine Ramathibodi Hospital, Mahidol University, Bangkok, THA; 2 Internal Medicine, Faculty of Medicine Ramathibodi Hospital, Mahidol University, Bangkok, THA

**Keywords:** adrenal gland neoplasm, area postrema, catecholamines, metabolic equivalent, vomiting

## Abstract

Pheochromocytomas are rare neuroendocrine tumors arising from chromaffin cells of the adrenal gland. Because of their highly variable clinical spectrum, these tumors often go undiagnosed and result in life-threatening complications. The typical presentations include episodic headache, palpitations and sweating accompanied with sustained or paroxysmal hypertension. However, less than half of pheochromocytoma patients have these classic symptoms. Many patients present with atypical symptoms, which could be overlooked. Our case represents an unusual presentation of pheochromocytoma, which is not well recognized as a possible manifestation. A 60-year-old woman presented with light-intensity-related nausea, which progressed to severe vomiting with hypovolemic shock. An unexpected adrenal mass was found during sonographic evaluation of the volume status. Pheochromocytoma was confirmed by 24-hour urine fractionated metanephrines and a computed tomography (CT) scan. In pheochromocytomas, the elevation of circulating catecholamines activates alpha-adrenergic receptors in the area postrema, which then initiates the emetic cascade. Light-intensity activity-related nausea and vomiting, especially when present with other symptoms of catecholamine excess, could be considered as a clinical presentation of pheochromocytomas.

## Introduction

Pheochromocytomas are catecholamine-secreting tumors derived from chromaffin tissues located in the adrenal medulla. The classic presentations of pheochromocytomas are episodic headache, palpitation and sweating associated with sustained or paroxysmal hypertension. However, most patients do not show this classic triad, and many may present with no clinical symptoms at all. Additionally, clinical manifestations of pheochromocytomas are highly variable and non-specific. Diagnosis of pheochromocytomas is therefore challenging and many cases are undetected, which results in improper treatment and devastating complications. In suspected cases, biochemical tests for excessive production of catecholamines and imaging studies are used to confirm the diagnosis and locate the tumor. Management of pheochromocytomas is based on appropriate preoperative medications and surgical removal. Long-term follow-up is required after surgery because of the risk of recurrence and metastasis [1].

The key to successful treatment is early suspicion of an existing tumor. We herein report light-intensity activity-induced nausea and vomiting, an unusual presentation of pheochromocytomas, which may help raise awareness of this rare disease.

## Case presentation

A 60-year-old high school teacher with a history of fairly-controlled diabetes and hypertension was brought to the emergency department because of severe nausea and vomiting with hypovolemic shock. For the past two days, she had experienced several episodes of non-bloody emesis without abdominal pain or diarrhea, which left her unable to eat. She had lost about 5 kilograms.

She recalled that, in a year, she had experienced nausea and palpitation a few times per month, which usually occurred after long talks during lectures. These symptoms were not associated with postural change. She once told her primary care doctor about them and had been told she might have anxiety. Typically, these irritating symptoms resolved after resting for minutes, but they had worsened over the previous two days.

Her initial vital signs were notable for a temperature of 38.3°C, heart rate of 114 beats per minute, and blood pressure of 88/54 mmHg. Laboratory examination revealed a white blood cell count of 9,300 cells/mm^3 ^withneutrophil 70%, lymphocyte 22%;^ ^hemoglobin of 13.8 g/dL and platelet count of 175,000 /mm^3^. Her serum creatinine was within the normal range (0.55 mg/dL). Liver function test results were as follows: alanine transaminase (ALT) 87 IU/L (normal, 0-55), aspartate transaminase (AST) 93 IU/L (normal, 5-34), alkaline phosphatase (ALP) 51 IU/L (normal, 40-150) and normal bilirubin levels.

**Figure 1:**
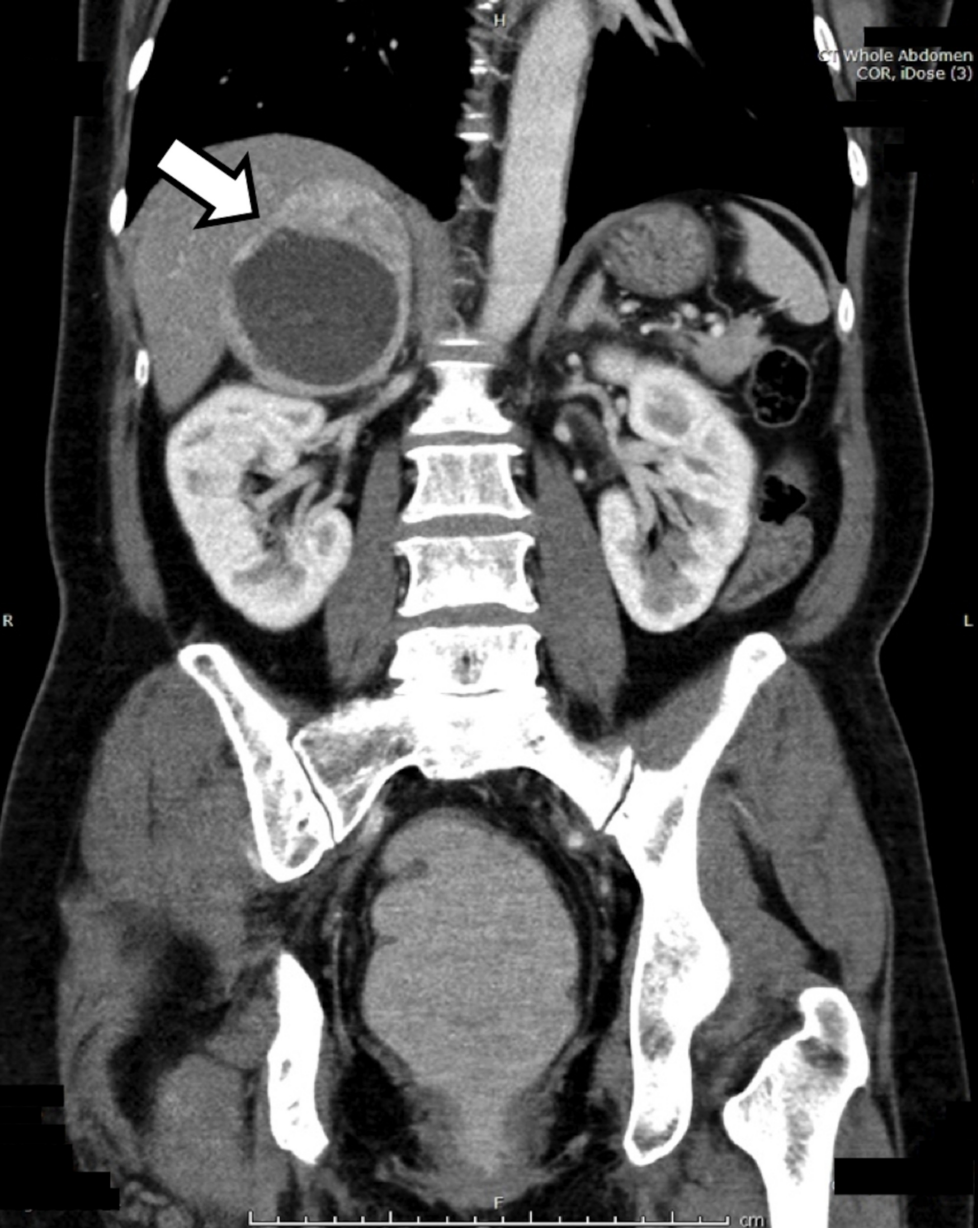
Computed tomography (CT) of the upper abdomen Contrast-enhanced coronal CT image demonstrates a large heterogeneously enhancing complex cystic-solid right adrenal mass.

After initial intravenous fluid resuscitation, her blood pressure increased to 180/110 mmHg. Inferior vena cava ultrasound was performed to assess her volume status. Incidentally, a heterogenous hypo/hyperechoic mass was found adjacent to the right kidney, which was initially misinterpreted as a liver abscess. She was sent for an abdominal computed tomography (CT) scan, during which an 8.0-cm heterogeneous enhancing complex cystic-solid mass with internal hemorrhage was located at the right adrenal gland (Figure 1). Pheochromocytoma was suspected, and alpha-blocker was started. Her symptoms dramatically improved on the same day and her blood pressure returned to normal. No family history of pheochromocytoma, paraganglioma or unexplained sudden death was reported.

**Figure 2:**
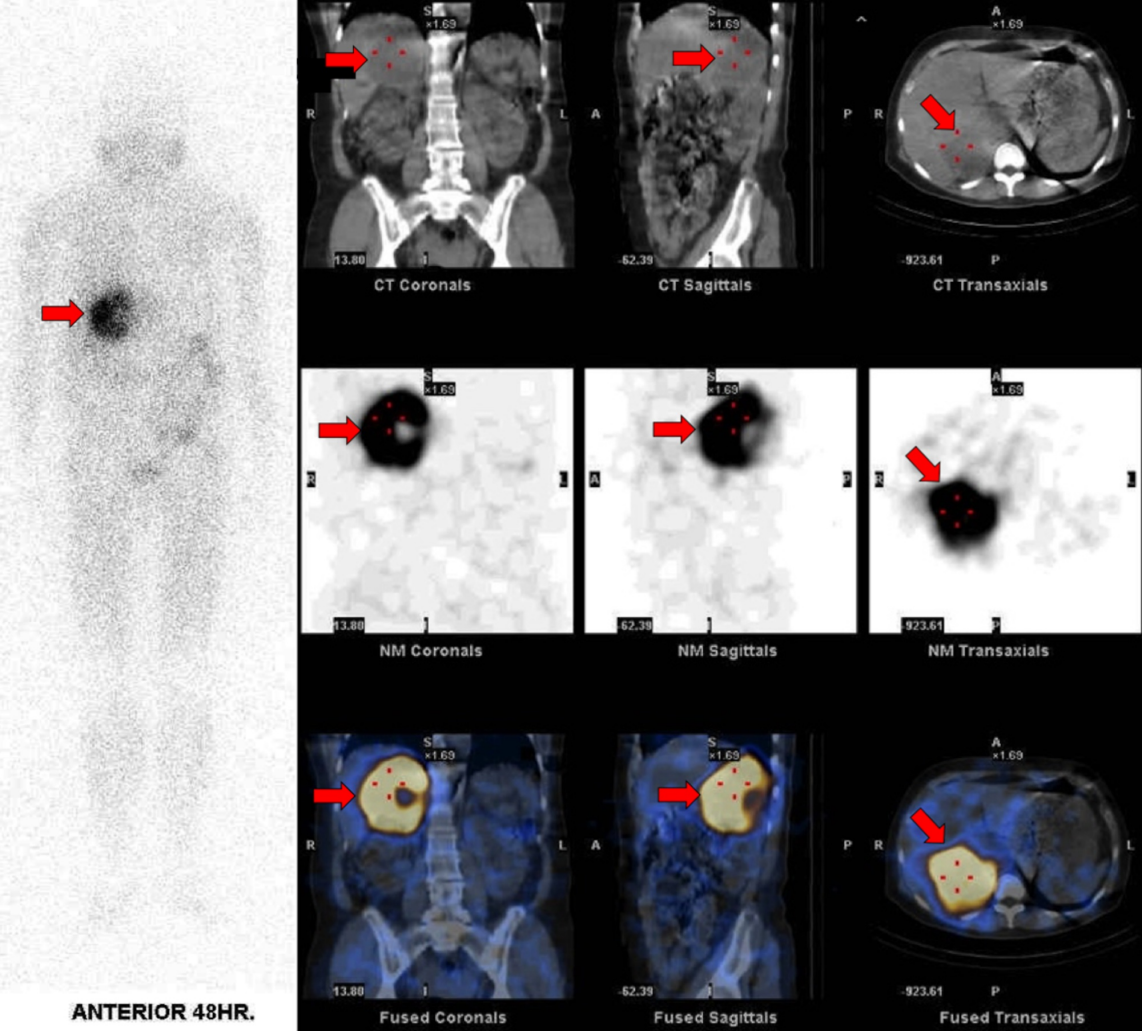
Meta-iodobenzylguanidine (MIBG) single photon emission computed tomography (SPECT/CT) MIBG SPECT/CT demonstrates avid unilateral radiotracer uptake in the large right adrenal mass (arrow), which is consistent with pheochromocytoma.

During admission, 24-hour urine fractionated metanephrines were collected. Revealed elevated urine normetanephrine were three times normal upper limits (normal value: <900 mcg/24 hour) and urine metanephrine 70 times the normal upper limits (normal value: <400 mcg/24 hour). Iodine meta-iodobenzylguanidine (MIBG) scintigraphy was arranged, which showed intense MIBG activity within the right suprarenal mass (Figure 2). After weeks of appropriate medical preparation, open adrenalectomy was successfully done. The patient was feeling well during a follow-up visit with no more event of episodic nausea. Her blood pressure was normotensive without any blood pressure-lowering drugs and her plasma glucose level was normal with no requirement of antihyperglycemic medications.

## Discussion

Clinical presentation of pheochromocytomas can vary from asymptomatic disease to severe hypertensive crisis. Signs and symptoms are mostly caused by excessive catecholamine production. Catecholamine stimulates three receptors throughout the cardiovascular system, alpha-adrenergic, beta-adrenergic and dopaminergic receptors; therefore, sympathetic activity would be intense. The well-known clinical triad of headache, tachycardia, and sweating are specific for this disease but not commonly found in most patients. Common signs and symptoms that can be seen in pheochromocytomas are headache (60%-90%), palpitations (50%-70%), sweating (55%-75%), pallor (40%-45%), nausea (20%-40%), weight loss (20%-40%), fatigue (25%-40%), anxiety and panic (20%-40%), flushing (10%-20%) and hyperglycemia (40%). Hypertension is also a common presentation. This can be classified into three forms, including sustained hypertension (50%-60%), paroxysmal hypertension (30%) and hypertension with orthostatic hypotension (10%-50%) [2]. Our patient's complaint of paroxysmal light-intensity activity (estimated metabolic equivalent level = 2.3) [3] related to nausea for a year. She overlooked this as a harmless gastrointestinal disturbance. This symptom progressed to severe nausea and vomiting with hypovolemic shock. However, after resuscitation, her blood pressure was unexpectedly high. We assume that during this episode of severe emesis, she also had hypertensive urgency. However, this was obscured by hypovolemic status.

**Figure 3:**
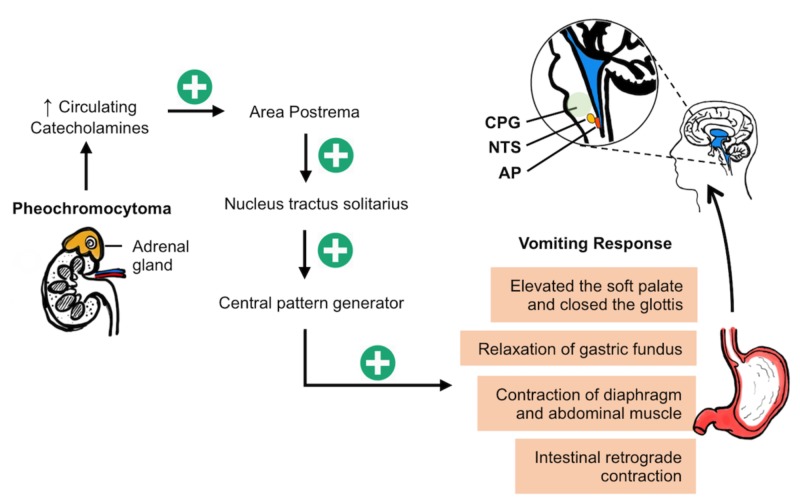
Vomiting cascade in pheochromocytomas In the hypercatecholamine stage of pheochromocytomas, increasing catecholamines directly activate an area postrema (AP) located at the caudal end of the fourth ventricle. This AP sends an emetic signal to nearby nucleus tractus solitarius (NTS,) which passes it to a central pattern generator (CPG). The CPG, defined as groups of neurons scattered throughout the medulla, ignites vomiting response by controlling a coordinated activity of the gastrointestinal tract, respiratory muscle, and muscle of the upper airway.

Sustained or paroxysmal hypertension is a typical presentation in pheochromocytomas. However, activity-induced nausea and vomiting are not well recognized as one of the possible manifestations. Physical activities such as lecturing, in this case, may stimulate catecholamine release from the tumor, which then triggers the emetic cascade. In the hypercatecholamine stage of pheochromocytoma, serum epinephrine and norepinephrine elevation activates alpha-adrenergic receptors in the area postrema, which triggers the emetic cascade (Figure 3) [4]. This area postrema, located at the floor of the fourth ventricle, serves as a chemoreceptor trigger zone for vomiting. Because there is no blood-brain barrier, this structure directly detects the circulating catecholamines [5].

Many studies, mainly in animals, support this mechanism of vomiting related to catecholamine response. Following an injection of the alpha-adrenoceptor agonists via the systemic or intracerebroventricular route, dogs and cats developed a vomiting response, which could be significantly suppressed by alpha-blockers [6-7]. Reports of postoperative nausea and vomiting in humans are substantially higher with general anesthesia agents that increase serum catecholamines such as cyclopropane and diethyl ether. Therefore, these agents are less commonly used in modern anesthetic practice [8-9].

A previous case series of nine people also found a similar association between physical activities and nausea in patients with pheochromocytomas [10]. Light to intense exercise stimulates nausea and vomiting in addition to the classic manifestations of the disease. The symptom ceased after removal of the primary tumor and recurred in some patients with metastatic lesions. The study also noted that 7.4% of succinate dehydrogenase subunit B (SDHB) related pheochromocytoma-paraganglioma developed exercise-associated symptoms while patients without lesions in the SDHB gene experienced only 1.6% of the symptoms. This could be explained by high catecholamines level, which is one of the characteristics in the SDHB phenotype. The phenotype also represents an aggressive behavior with high malignant potency and metastatic rate [10]. Unfortunately, genetic testing was not performed in our patient. However, no metastasis was detected by MIBG scan and no recurrence of the tumor was found at three years after surgery.

## Conclusions

We suggest that light-intensity activity-related nausea and vomiting, especially when accompanied by other symptoms of catecholamine excess, should be considered as one of the clinical presentations in pheochromocytomas and may help to determine the cause of these mysterious tumors.

## References

[1] Plouin PF, Amar L, Dekkers OM (2016). European Society of Endocrinology Clinical Practice Guideline for long-term follow-up of patients operated on for a phaeochromocytoma or a paraganglioma. Eur J Endocrinol.

[2] Lenders JW, Eisenhofer G, Mannelli M, Pacak K (2005). Phaeochromocytoma. Lancet.

[3] Ainsworth BE, Haskell WL, Herrmann SD (2011). 2011 compendium of physical activities: a second update of codes and MET values. Med Sci Sports Exerc.

[4] Hornby PJ (2001). Central neurocircuitry associated with emesis. Am J Med.

[5] Jenkins LC, Lahay D (1971). Central mechanisms of vomiting related to catecholamine response: anaesthetic implication. Can Anaesth Soc J.

[6] Hikasa Y, Ogasawara S, Takase K (1992). Alpha adrenoceptor subtypes involved in the emetic action in dogs. J Pharmacol Exp Ther.

[7] Jovanovic-Micic D, Samardzic R, Beleslin DB (1995). The role of alpha-adrenergic mechanisms within the area postrema in dopamine-induced emesis. Eur J Pharmacol.

[8] Palazzo MGA, Strunin L (1984). Anaesthesia and emesis I: etiology. Can Anaesth Soc J.

[9] Black GW, McArdle L, McCullough H, Unni VK (1969). Circulating catecholamines and some cardiovascular, respiratory, metabolic and pupillary responses during diethyl ether anaesthesia. Anaesthesia.

[10] King KS, Darmani NA, Hughes MS, Adams KT, Pacak K (2010). Exercise-induced nausea and vomiting: another sign and symptom of pheochromocytoma and paraganglioma. Endocrine.

